# Identification of sensorineural hearing loss subtypes using unsupervised machine learning and assessment of their replicability

**DOI:** 10.1038/s41598-025-33815-9

**Published:** 2026-01-20

**Authors:** Lilia Dimitrov, Watjana Lilaonitkul, Nishchay Mehta

**Affiliations:** 1 ENT Department, Royal National Throat Nose and Ear Hospital, London, UK; 2 evidENT UCL Ear Institute, University College London, London, UK; 3 National Institute of Health Research University College London Hospitals Biomedical Research Centre, London, UK; 4 Medical Research Council, London, UK; 5 Global Business School of Health, University College London, London, UK

**Keywords:** Diseases, Computer science

## Abstract

Despite nearly 20% of the global population experiencing hearing loss, there remains limited insight into the underlying subtypes of its most prevalent cause, sensorineural hearing loss (SNHL). This understanding is crucial for effective therapeutic and preventative strategies. A recent study using a Gaussian Mixture Model (GMM) identified 10 distinct SNHL phenotypes in a large US cohort, highlighting the potential of unsupervised machine learning (UML) to provide a data-driven solution to this task. Rigorous validation of these models is essential; however, it is limited due to several factors, including the absence of ground truth labels for model evaluation, restricted data access, and the lack of a standardized reporting framework for comparing results across clustering studies. Here, we apply a GMM to a UK database of 109,854 audiograms, revealing 9 phenotypes, partly overlapping with prior findings. Notably, our study cohort is characterized by advanced age, a higher proportion of female participants, and more severe hearing impairments. We observed instability in the GMM model when subjected to variations in the original dataset. To enhance practices, we propose a Clustering Replicability Framework, ensuring robustness in UML driven health research for safe clinical translation.

**Supplementary Information:**

The online version contains supplementary material available at 10.1038/s41598-025-33815-9.

## Introduction

Globally 1.5 billion people have hearing loss, making it the most common sensory disorder worldwide^1^. The risk of developing sensorineural hearing loss (SNHL), the most common form of adult hearing impairment, increases with age and therefore the burden of hearing loss is predicted to rise further as our aging population increases. Hearing loss has a substantial impact on quality of life, cognition, and communication, as well as being identified as a key modifiable risk factor for developing dementia^2–4^. The delivery of hearing care is also placing a huge demand on increasingly stretched public health services. In 2010-11 alone the estimated cost of managing hearing loss in England was £450 million^5^.

Despite this, practical treatments for SNHL have seen minimal evolution since the introduction of the analogue hearing aid in the 1970s. Cochlear implants and novel gene therapies for monogenic hearing loss are notable exceptions however they are limited to only a very narrow subset of the patient population^6^. Recent advances in understanding the genetic and molecular pathways associated with SNHL have spurred the development of novel drug, gene, and cell therapies, showing promising results in reversing more common causes of SNHL (including age-related, noise-induced and ototoxic drug-induced hearing loss) in animal models^7–9^. However, the translation of these therapies to successful clinical trials in humans has been hindered, in part, by the challenge of identifying the specific patient populations that stand to benefit from these highly targeted treatments. Better phenotyping of SNHL is essential for the progress of new treatments^7^.

Identifying the underlying pathology in the more common causes of adult SNHL can currently only be performed post-mortem which is of limited utility. Although there is work to identify circulating biomarkers for hearing loss, this is still in its infancy^10,11^. The gold-standard assessment and most widely performed investigation for hearing loss is pure tone audiometry (PTA). As such, this non-invasive and ubiquitous investigation has great potential as a candidate biomarker to infer underlying subtypes. Linking audiograms and underlying pathology is not novel. It was first recognized in the 1930’s through the creation of an age-related hearing loss classification system that mapped lesion site to audiogram configuration, identifying four primary subtypes: sensory, neuronal, strial/metabolic, and cochlear^12–14^. 

Since then, various methods have been employed for identifying distinct audiogram-defined types, encompassing approaches from expert consensus^15,16^ to quantitative methodologies^17–19^. One emerging approach to this problem has been to harness unsupervised machine learning (UML) methods to understand audiogram heterogeneity in a small number of studies^19–23^. UML methods identify high sample densities in datasets without imposing any prior knowledge or classification systems. This approach is particularly valuable in light of recent challenges to traditional audiogram-pathology associations, prompted by advances in our understanding of the molecular and genetic mechanisms underlying hearing loss. For instance, limitations in techniques to measure hair cell survival may have underestimated the role of hair cell loss in presbycusis^24^. Additionally, a study across 160 temporal bones challenges the conventional belief that strial atrophy correlates with audiogram flatness^25^. 

Unlike supervised learning, where machine learning models are trained on labelled data and evaluated based on their ability to predict those labels accurately, unsupervised learning deals with unlabelled data. As a result, evaluation metrics used in supervised learning, such as accuracy, precision, recall, and F1-score, are not directly applicable in the unsupervised learning context. Since there are no ground truth labels to compare the model’s output against, evaluating the performance of UML becomes inherently challenging. Replication provides a mean for assessing the stability of these models both within and across datasets.

A recently published paper applied a gaussian mixture model (GMM) to 132,504 audiograms from the Massachusetts Eye and Ear (MEE) database and identified 10 audiogram clusters, with a smaller number of 6 clusters identified within an additional publicly-available normative database^19^. GMM has the benefits that it can decompose any dataset into a combination of a set number of gaussian components without any a priori knowledge. It has the advantage over models such as K-Means, which have been used to address the identification of audiometric clusters in a few studies because it does not assume sphericity of clusters^20,26^. GMM is however very sensitive to initialisation methods and requires specification of the number of optimum clusters which can be done using several methods. Both factors pose potential issues for the reliability of results across different settings.

The GMM study by Parthasarathy et al.^19^ involves a similar clinical setting to our own institution and the only comparably sized dataset in the published literature. As such, the opportunity presents to evaluate the clinical validity of their model by applying it to a similar hearing health population within the United Kingdom (UK). We extend this work by employing quantitative methods to evaluate cluster replicability within our own dataset to assess the overall stability of the GMM model. Finally, to facilitate comparability between future health data clustering studies, we make a novel contribution in the form of a Clustering Replication Framework.

## Results

### Study population

**Fig. 1:**
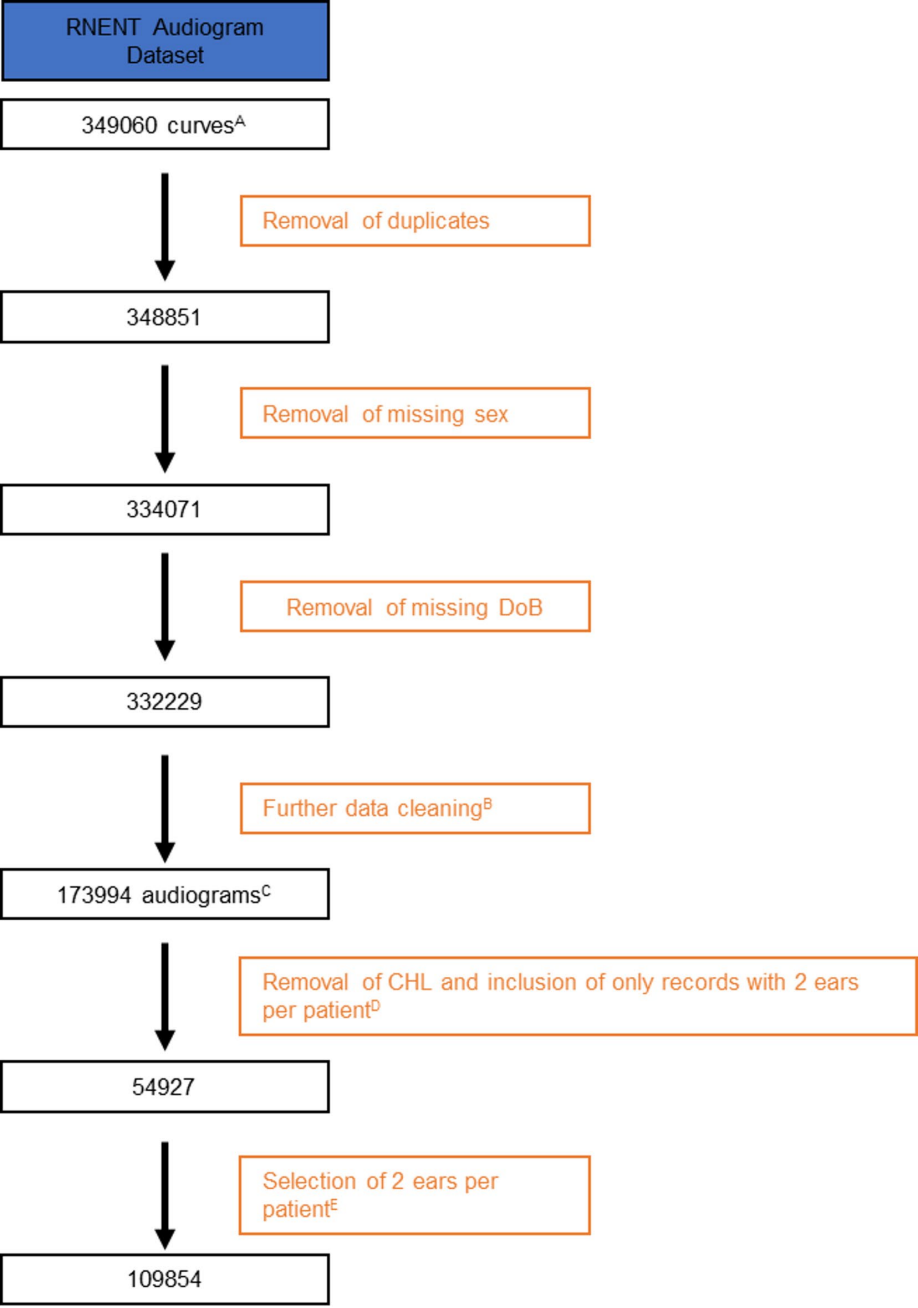
Study flow diagram for inclusion of records into the study. ^A^Data Curves. In Auditbase, audiogram data is stored in a tabular format, where each row represents a set of thresholds collected for a patient using a specific testing modality. Audiograms can be conducted by measuring air-conduction (AC) thresholds alone—where pure tones are delivered via headphones—or AC and bone-conduction (BC) thresholds, the latter involving tones played to the temporal bone. When both AC and BC thresholds are measured during the same test for a given patient, Auditbase saves them as two separate records, referred to as *curves*. These curves are subsequently joined to reconstruct a single audiogram that includes both AC and BC thresholds. ^B^Further data cleaning. This is detailed in full in the Supplementary Fig. 1 and in Appendix 4. In summary of order of processes: (1) Audiogram curve records with no threshold values were removed. (2) Duplicated audiogram curve records — defined as records indexed as separate audiograms but conducted on the same day with the exact same thresholds — were removed. (3) In cases where multiple, non-duplicate audiogram curve records existed for the same day, a single audiogram curve was included (the rule used to determine which curve was kept is outlined in Appendix 4 of the supplementary information). (4) Records which had threshold values outside of the range of testing (−10 to 120dB) or that were not in multiples of 5 were removed. ^C^The records are now referred to as audiograms. After performing data cleaning, records with both AC and BC curves were joined to create a single row per audiogram. Some audiograms will be a single AC curve and some will be 2 curves if they had AC and BC performed. This is why the number of audiograms drops in the table at this point. ^D^CHL: Audiograms displaying conductive hearing loss are removed. Records where each patient has SNHL in both ears are included only. Only records from patients aged ≥ 18 are included. The values for both ears are stored in a single row per patient. Superscript E: Each ear is treated as an independent observation and is stored in its own row. This is why the number of audiograms doubles in the final step.

Our final dataset (referred to as the RNENT dataset from here onwards) included 109854 audiograms from 54,927 patients between 1981 and 2021 (Fig. 1). Please see supplementary materials for a detailed breakdown of how the final dataset was achieved (appendices 1–4 and Supplementary Figure).

The summary statistics for the RNENT dataset are displayed alongside the available data for the comparator MEE study in Table 1.

**Table 1:** Summary of patient characteristics in the Royal National Ear, Nose and Throat dataset (*N* = 54927) in comparison with the Massachusetts Eye and Ear dataset (*N* = 116400). The demographics are presented for the entire database *N* = 116400 patients, however of these, only 66252 patients had audiograms used in the GMM model. The demographics of this subcohort are not presented in the published study so we present the demographics of the entire dataset only.

	RNENT (*N* = 54927)	MEE (*N* = 116400)
**Age (years)**		
Mean (SD) Range Proportion ≥ 50 years (%)	61.9 (± 19.7) 18–100 71	- 18–80 63
**Gender (%)**		
Male Female	44 56	46–49 as function of age group 51–54 as function of age group
**GMM** ^**a**^ **model parameters**		
Cluster number Covariance Type Regularisation Value Convergence Thresholds	9 Full 0.01 le-3	10 Full 0.01 le-6

^a^ GMM, Gaussian Mixture Model.

**Fig. 2:**
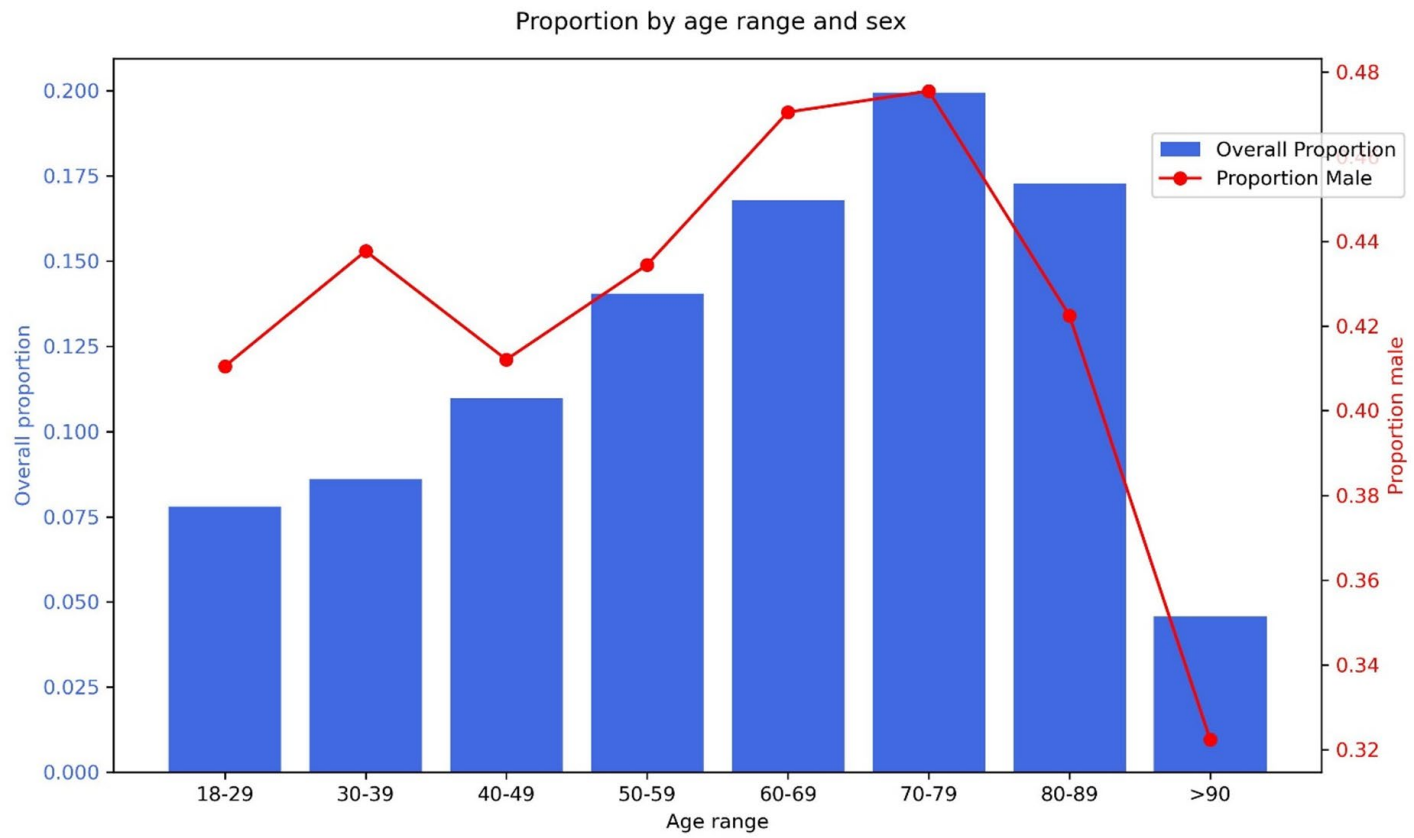
Distribution of patients across age ranges (blue bars) with superimposed proportions of male sex (red line), *N* = 54927.

The modal age range of the RNENT dataset was 70–79 years (Fig. 2). This is older than the MEE dataset which had a bi-modal age range of 50–59 and 60–69. 21.9% of the RNENT sample were 80 years or older (the maximum age for inclusion was 80 years of age in the MEE study).

56% of patients were female (Table 1). Sex distribution was broadly stable across the different age ranges below 80 years (range of male: 41–48%) but there was a marked drop in proportion of males in patients over 90 (Fig. 2). This mirrors known trends in sex-differences in life expectancy in the UK^27^. The MEE dataset did not contain patients from the two upper ages ranges present in the RNENT study but the proportions of male patients was higher overall and ranged between 46–49% across all other age ranges.

### GMM-identified audiogram subtypes

**Fig. 3:**
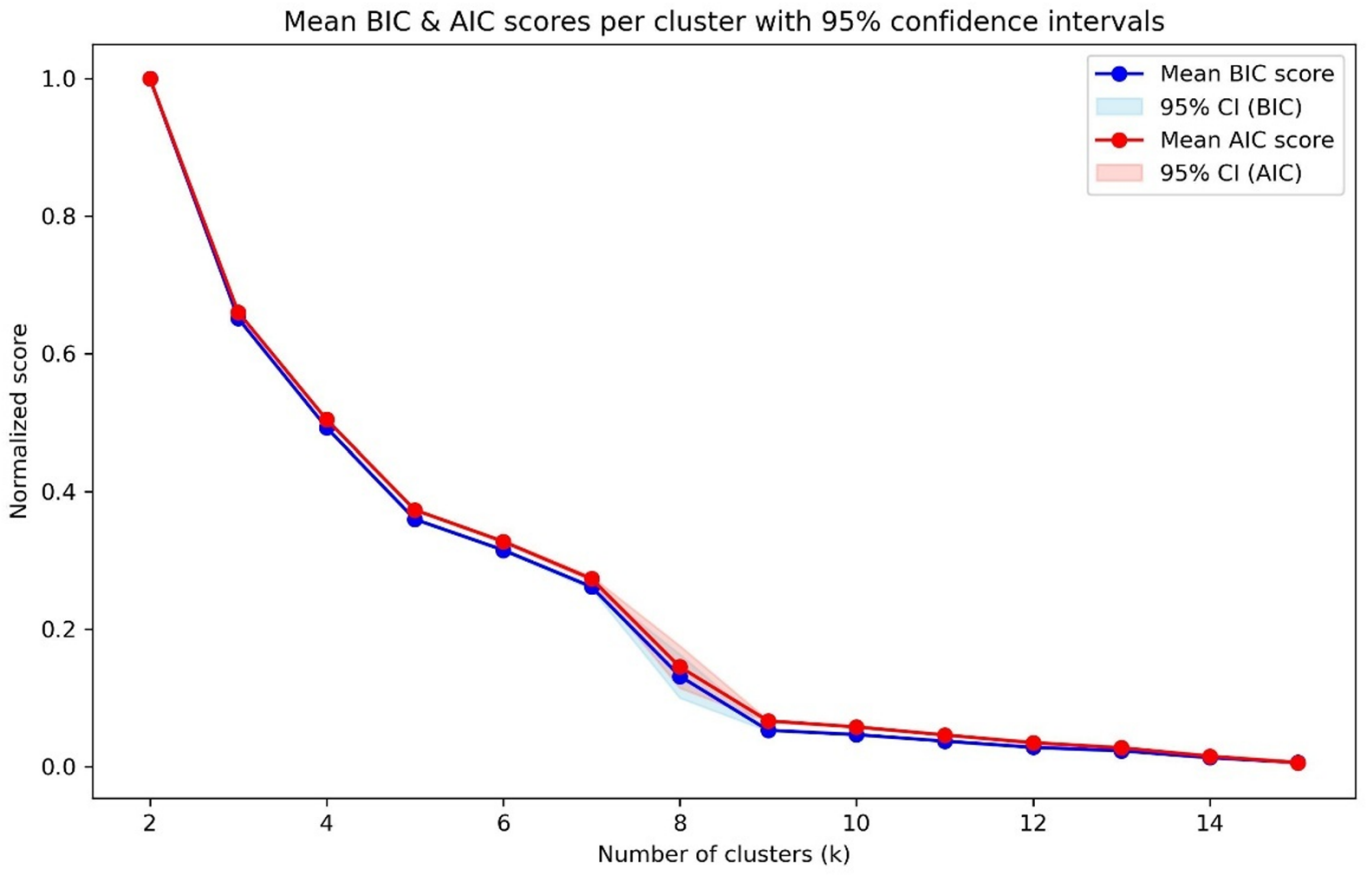
Bayes Information Criterion (BIC) and Akaike Information Criterion (AIC) values for each cluster number. The blue and red lines indicate respectively the mean BIC and mean AIC across the 21 different random seeds. The pale blue and pale red shaded areas respectively represent the corresponding 95% confidence intervals, *N* = 109854.

Mean Bayesian information criterion (BIC) and Akaike information criterion (AIC) were calculated across 2–15 clusters with 21 random seeds per cluster yielding a total of 294 combinations. Mean BIC and AIC scores as a function of cluster number are displayed in Fig. 3. The mean BIC and AIC values reach a plateau at 9 clusters. In the MEE study, the BIC curve also plateaued rather than demonstrating a clear elbow, but this was found at 10 clusters^19^.

**Fig. 4:**
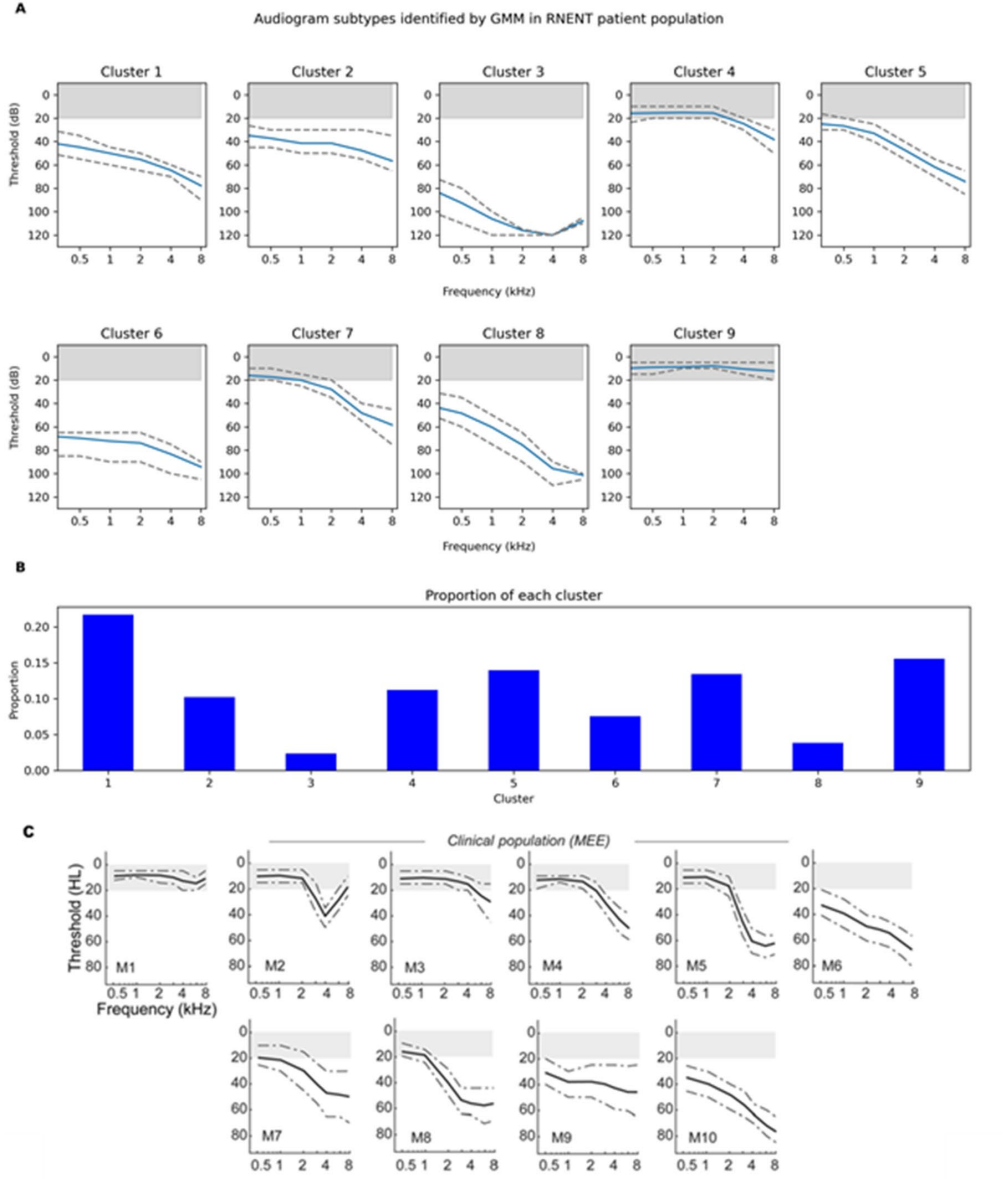
**(A)** Gaussian Mixture Model (GMM) identified audiometric subtypes using Royal National Ear, Nose and Throat (RNENT) dataset. Blue solid lines indicate the mean thresholds per frequency and dashed lines represent the upper quartile range and lower quartile values for each frequency. The grey-shaded bar at the top of each graph represents the normal thresholds of hearing. **(B)** Bar chart showing the proportion of records belonging to each subtype in the dataset, N = 109854. (**C**) Gaussian Mixture Model (GMM) identified audiometric phenotypes using Massachusetts Eye and Ear (MEE) dataset. Grey solid lines indicate the means and dotted lines indicate the interquartile range (50%), N = 132,504). Note the y-axis label “HL” corresponds to decibel (dB) hearing level which is the equivalent to dB (decibels). Printed with permission from corresponding author.

The 9 audiogram profiles associated with each cluster are shown in Fig. 4A with the corresponding proportions of each patient in Fig. 4B. Figure 4C depicts the 10 audiogram clusters identified in the comparator MEE study and is included for reference for the reader.

**Fig. 5:**
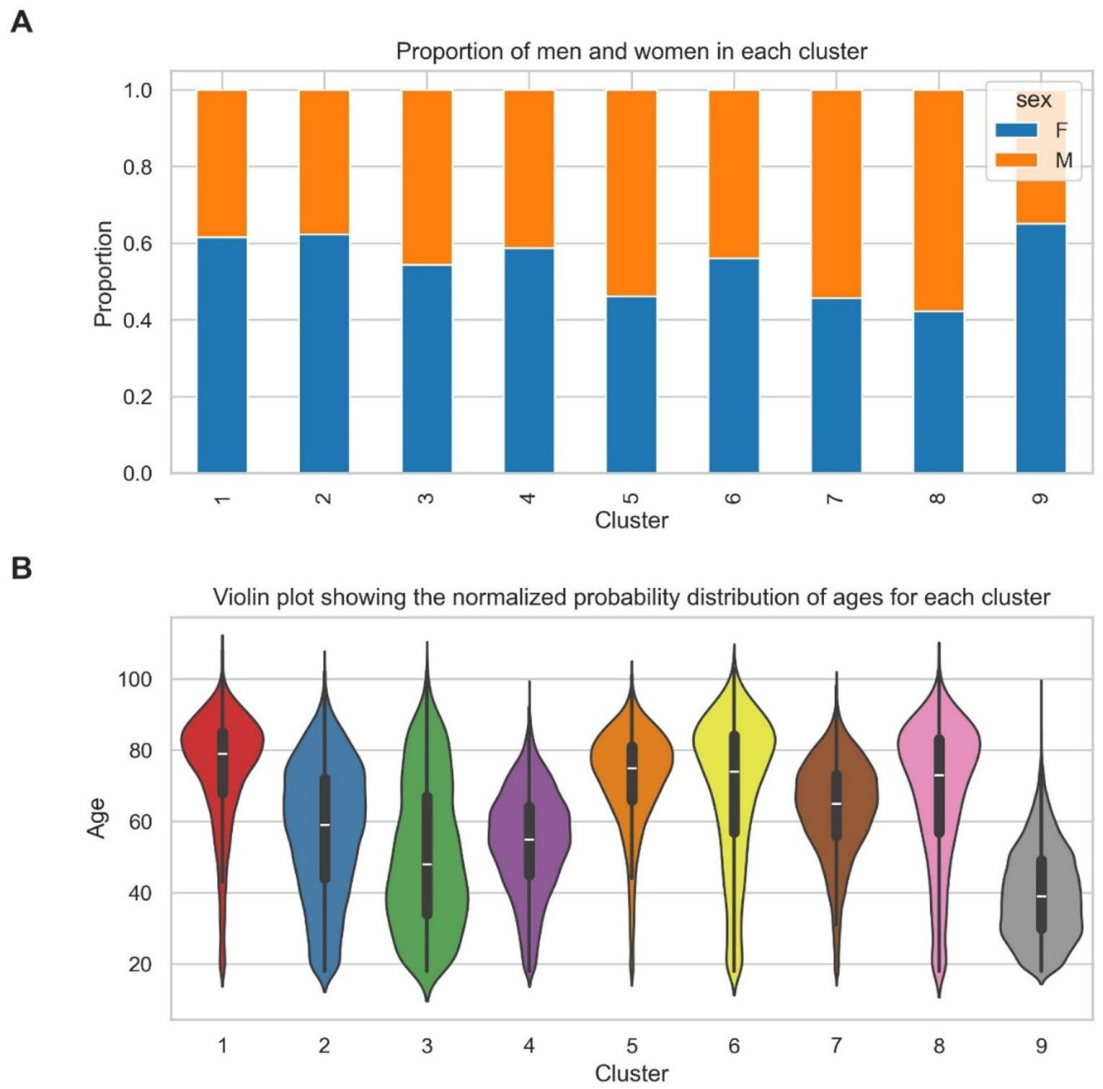
**(A)** Stacked bar chart visualising proportion of men (M, orange bars) and women (F, blue bars) per subtype. **(B)** Violin plot visualising age distribution per subtype. The median values for age are represented by a white dot, the 1 st and 3rd quartile by the lower and upper limits of thick central bar and the minimum and maximum age values indicated by the lower and upper limit of thin central line.

Violin plots were used to visualise the relationship between cluster type and age (Fig. 5B). As demonstrated, clusters 9, 3 and 4 are associated with the lowest median age whereas clusters 1, 5, 6 and 8 with the highest. In general, most clusters were over-represented by women except for clusters 5, 7 and 8 (Fig. 5A).

The shapes of our clusters qualitatively overlapped with those from the MEE study however there are some key differences (Table 2). Overall, the audiogram profiles from our analysis are associated with worse hearing thresholds, with fewer profiles falling within the normal thresholds for hearing (shaded grey areas in Fig. 4A) (only Cluster 4, 7 and 9). Broadly, there are 5 groups of profiles. Subtype 9 represents normal hearing, Subtypes 4 and 7 exhibit patterns consistent with presbyacusis with normal low-frequency hearing with varying degrees of high-frequency hearing loss, Subtypes 5 and 6 show a flatter hearing loss profile in the low frequencies with down sloping hearing loss in the higher frequencies, whereas subtypes 1, 2, and 8 display down-sloping hearing loss across all frequencies. Finally, Subtype 3 demonstrates severe-to-profound hearing loss across all frequencies with a 4 kHz notch. We did not identify the more typical notched audiogram found by the MEE study (Fig. 4A, M2), which is characterised by a downward inflection at 4 kHz but at lower thresholds and is associated with early noise-induced hearing loss (NIHL).

Our dataset had 2 audiogram profiles with high thresholds across all frequencies (clusters 3 and 6) which were not well matched to any of the audiograms in the MEE study. Conversely, our audiogram profiles did not demonstrate the pattern of normal low frequency thresholds with moderate-severe hearing loss at higher frequencies as found in the MEE study (clusters M5 and M8).

**Table 2:** Qualitative comparison between the different clusters identified in the Royal National ear, nose and throat (RNENT) and Massachusetts eye and ear (MEE) datasets. Clusters with similar audiogram profiles are described as overlapping. Audiogram profiles with no clear counterpart in the other dataset are described as not overlapping. M is the prefix used to identify the different clusters in the MEE dataset.

	RNENT cluster	MEE cluster
	Cluster allocation	
Overlapping	Cluster 1	M6
	Cluster 2	M9
	Cluster 4	M3
	Cluster 5	M7
	Cluster 7	M4
	Cluster 8	M10
	Cluster 9	M1
Not overlapping	Cluster 3	-
	Cluster 6	-
	-	M2 (notched)
	-	M5
	-	M8

### Noise-induced hearing loss phenotype subsumed into other clusters

**Fig. 6:**
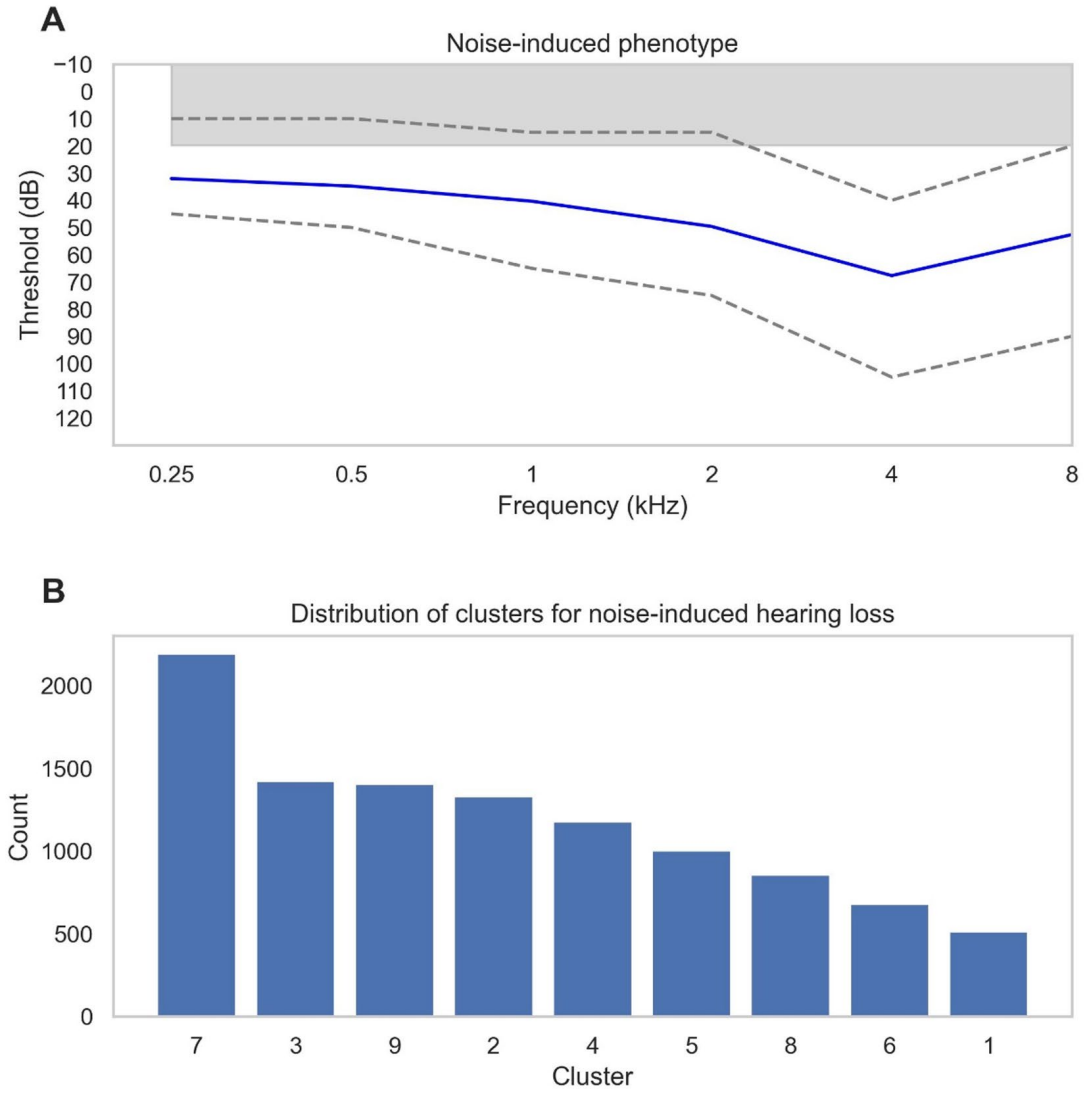
**(A)** Audiogram profile of all audiograms within the Royal National Ear, Nose and Throat (RNENT) dataset with an audiometric notch defined as at least a 10dB increase in hearing threshold at 4 kHz compared to 1–2 kHz and at least a 10dB increase in hearing threshold at 4 kHz compared to at 8 kHz using Coles’ criterion^28^. Solid blue lines indicate the mean threshold per frequency and grey dashed lines represent the upper quartile range and lower quartile values for each frequency. The grey-shaded bar at the top of each graph represents the normal thresholds of hearing. *N* = 10,580. **(B)** Bar chart showing which clusters the audiograms with an audiometric notch were assigned to by the Gaussian Mixture Model. The bars are arranged in order of frequency.

There were 10580 audiograms who met Coles’ criterion for NIHL in our dataset (Fig. 6A) but our GMM model did not identify the classical NIHL audiometric phenotype found in the MEE study (Fig. 4A, M2). The patients with the NIHL notch were mainly subsumed by cluster 7 and then almost evenly across clusters 3, 2 and 9 (Fig. 6B). Cluster 7 represents normal hearing thresholds at low frequencies with reduced thresholds at higher frequencies. Cluster 3 demonstrates the inflection point at 4 kHz but the hearing thresholds are much more severe than typically seen in NIHL. Cluster 9 is normal thresholds whereas cluster 2 shows a flat mild-moderate hearing loss profile.

Components of all these profiles therefore overlap with the NIHL audiogram which typically displays normal low frequencies and worse high frequencies but the characteristic notch at 4 kHz has not been isolated as a separate cluster. Looking at the audiogram profile of all the patients identified with NIHL in the dataset based on audiometric criteria (Fig. 6A), it is apparent that at both 4 kHz and 8 kHz there is a wider spread of thresholds than at other frequencies. This variance could explain why our model could not isolate the inflection amongst the noise.

### Reverse-loss hearing phenotype subsumed into other clusters

**Fig. 7:**
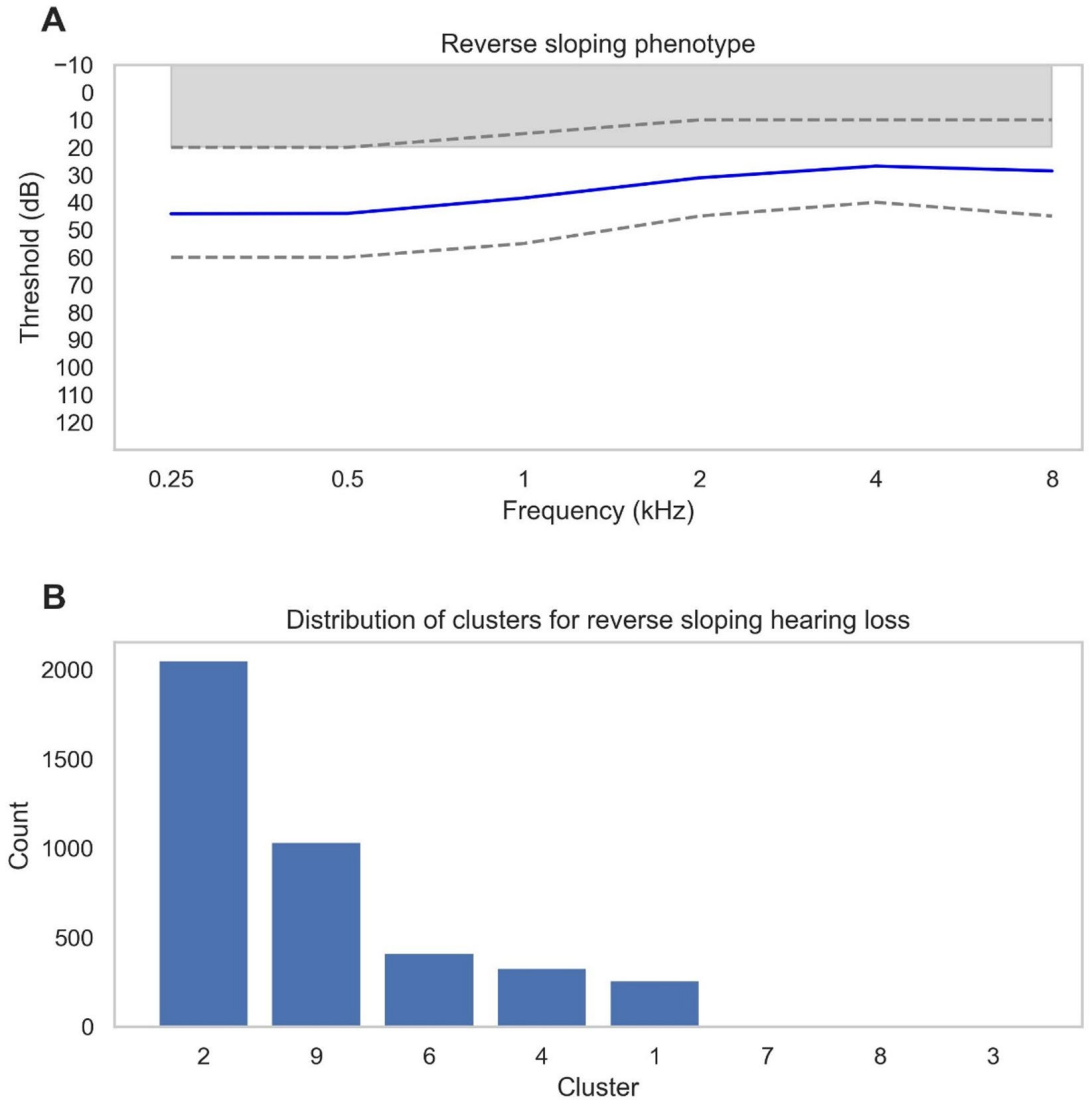
**(A**) Audiogram profile of all audiograms within the Royal National Ear, Nose and Throat (RNENT) dataset with reverse slope hearing loss defined as an average of 10dB of more lower thresholds in the high frequencies (4 and 8 kHz) compared to the low frequencies (0.25 and 0.5 kHz)^29^. Solid blue lines indicate the mean threshold per frequency and grey dashed lines represent the upper quartile range and lower quartile values for each frequency. The grey-shaded bar at the top of each graph represents the normal thresholds of hearing (*N* = 4107). **(B)** Bar chart showing which clusters the audiograms with a reverse sloping profile were assigned to by the Gaussian Mixture Model. The bars are arranged in order of frequency.

There were 4107 audiograms where the mean high frequency thresholds (4 and 8 kHz) were 10 dB or more lower than the mean low frequency thresholds (0.25 and 0.5 kHz) (Fig. 7A)^29^. Most of these cases were classified into Cluster 2, followed by Cluster 9, which represents normal hearing (Fig. 7B).

One possible explanation is that the audiometric curve shape of the reverse-slope phenotype is relatively flat, resembling the profiles seen in Clusters 2 and 9. Although reverse-slope loss is defined by its characteristic upward-sloping pattern, the overall curvature remains less steep compared to other hearing loss configurations, making it more difficult for the model to differentiate. As a result, these patients were largely absorbed into Cluster 2, which appears to represent a broad, intermediate category of mild-to-moderate hearing loss with a relatively flat shape.

Another contributing factor is the high variability in low-frequency thresholds among patients with Ménière’s-like audiograms, as indicated by the broader error bars at these frequencies in Fig. 7A. This within-group variability weakens the ability of the clustering algorithm to recognize these patients as a cohesive, distinct phenotype, leading them to be distributed across other clusters instead. Additionally, at higher frequencies (2–8 kHz), the thresholds of the reverse-slope group overlap significantly with normal hearing levels, further contributing to their misclassification into Cluster 9. This overlap may explain why these patients are not grouped separately but rather distributed between normal hearing (Cluster 9) and the broad flat-loss group (Cluster 2).

### Symmetry between ears

**Fig. 8:**
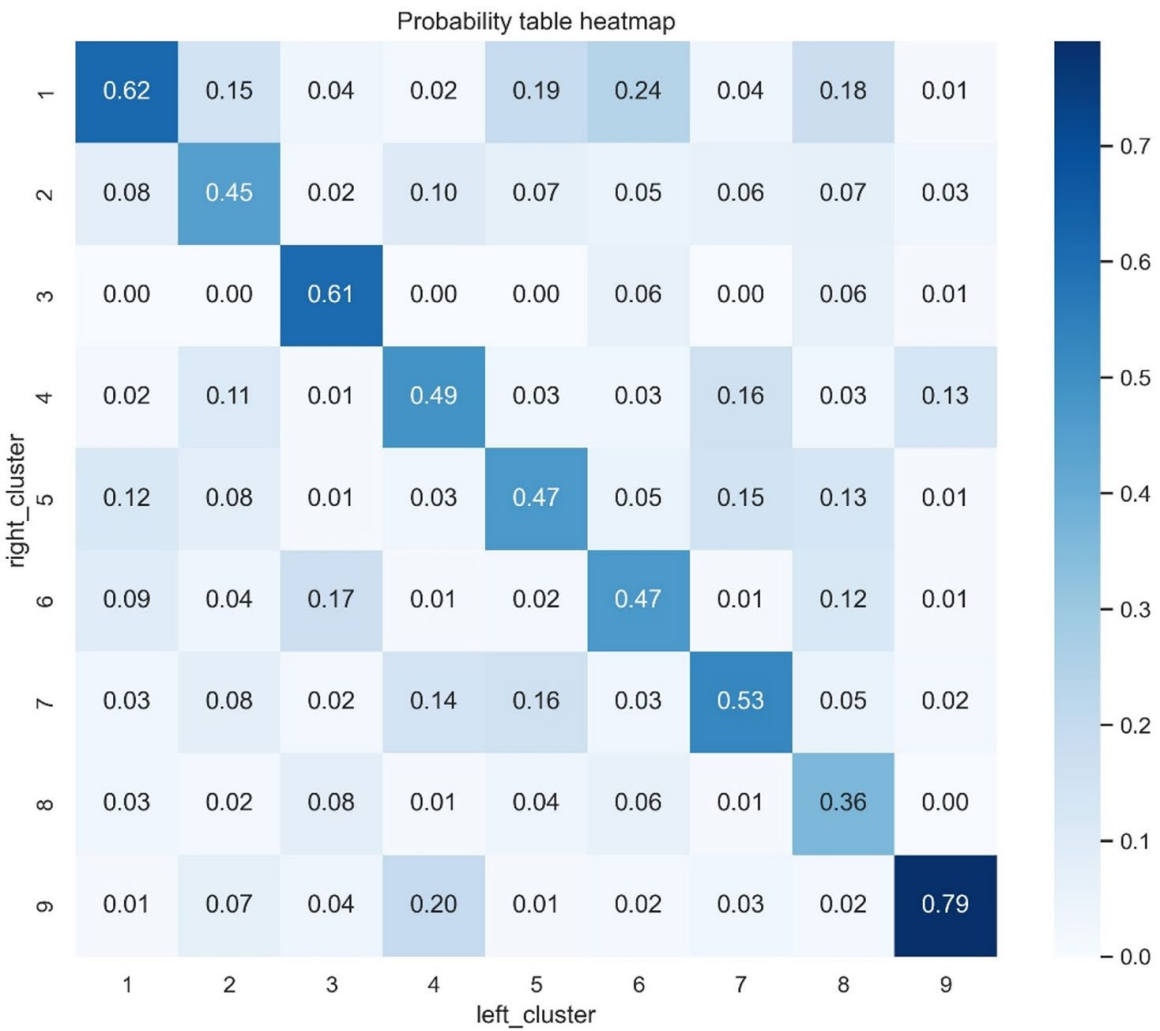
Heat mapping showing probability of cluster type in the right ear given cluster type in the left ear. Darker blues indicate greater percentage of occurrence. The probabilities are shown in each box rounded to 2 decimal places. The heat map is designed to be read vertically.

To assess the symmetry of cluster assignment between ear pairs, a contingency table was created and the conditional probabilities of right ear cluster assignment given the left cluster value of were plotted as a heat map (Fig. 8, to be read vertically). There was strong relationship between the cluster assignments of the ears as demonstrated for cluster 9 (normal hearing), cluster 3 (the most severe hearing loss phenotype) and cluster 1.

### Replication analysis

**Fig. 9:**
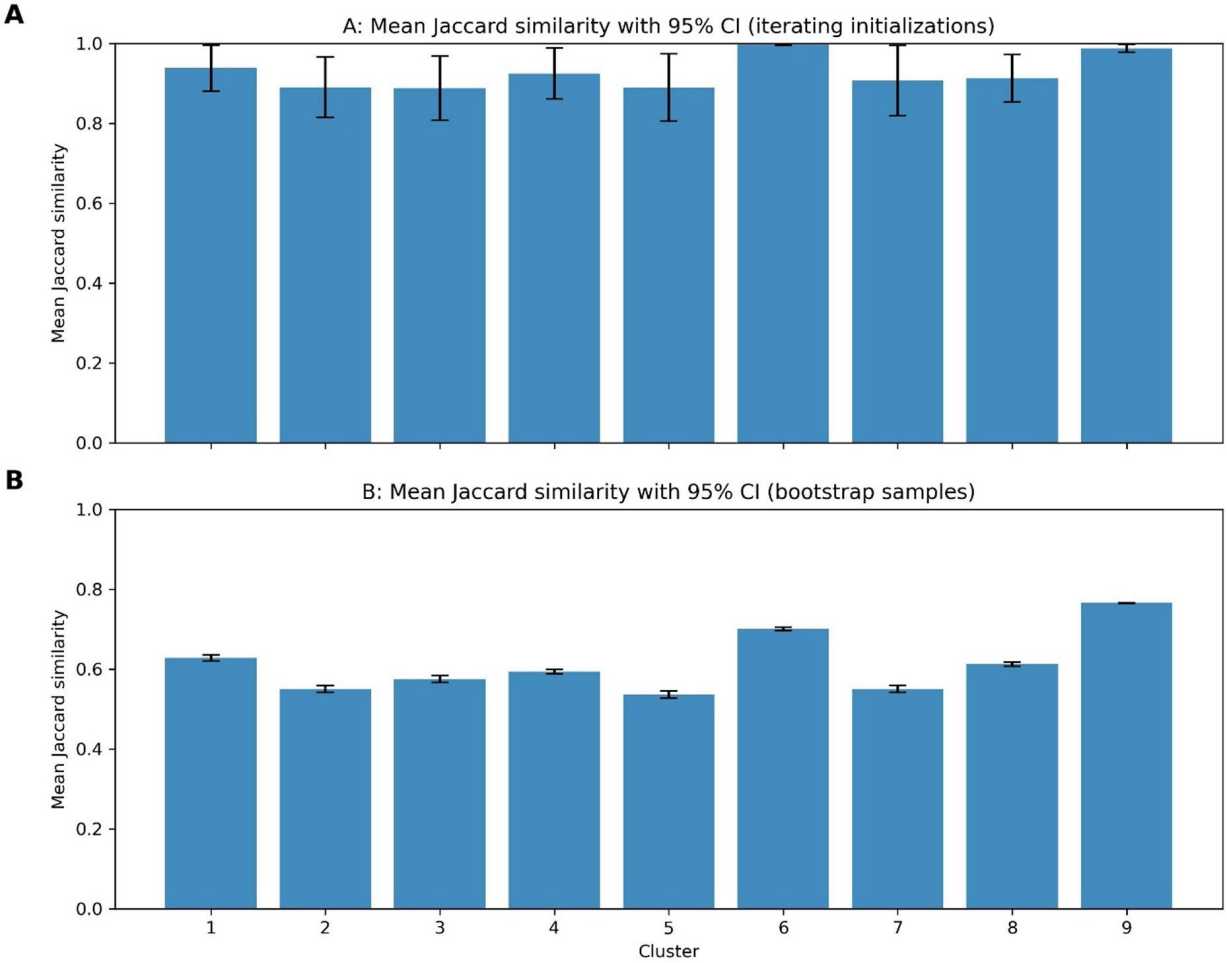
Mean Jaccard Similarity scores across the maximum Jaccard similarity scores observed within each cluster. These scores are calculated in two contexts: across different initializations (A) and across different bootstrap samples (B). The comparison is made with the original model output clusters. Error bars represent the 95% confidence intervals.

The clusters from the original dataset were generally found poorly by the GMM across the bootstrap samples with a range of Jaccard scores 0.59–0.69 with the exception of cluster 9 (normal hearing group) which had the highest Jaccard score of 0.77 (Fig. 9B). Performance across different initialisations performed well with high levels of cluster replicability found for all clusters across the 21 different initialisations (Fig. 9A). This suggests that initialisation parameters do not impact the model performance significantly but slight alterations in the dataset can lead to identification of different clusters.

**Fig. 10:**
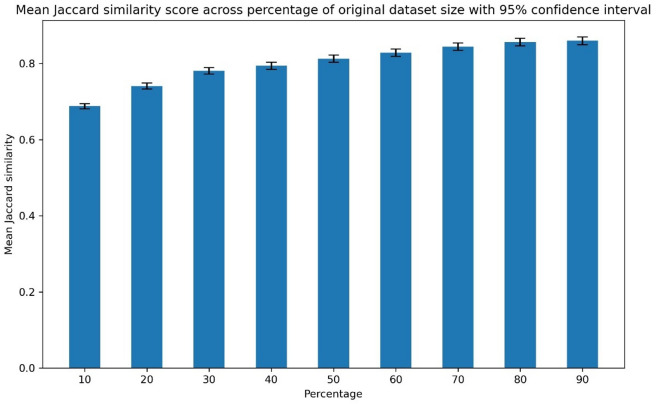
Mean Jaccard similarity score across different sizes samples ranging between 10–90% of the original dataset size (*n* = 109854). Bar charts display the mean Jaccard similarity score across 1000 samples per percentage of the original dataset. 95% confidence intervals are represented by the error bars.

Performance, using the Jaccard score, whilst clustering across different sized samples is depicted in Fig. 10. A sample size of at least 50% of the original dataset (*n* = 109854) is required to achieve a Jaccard score above 0.8.

### Clustering replication framework

**Fig. 11:**
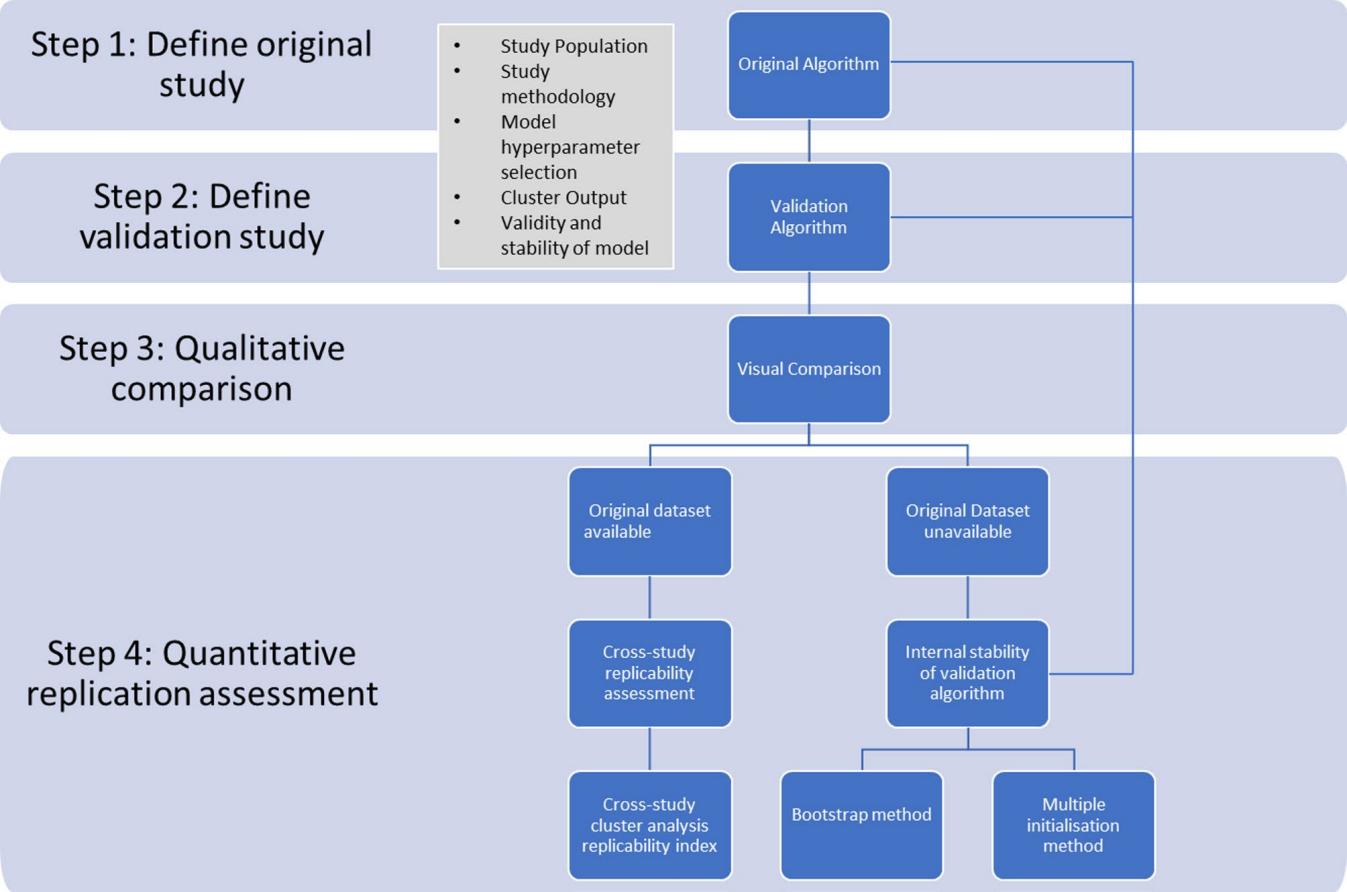
Clustering replication framework. Suggested framework to guide future clustering replication studies. Validation algorithm refers to the model being used by the investigators replicating the original study.

The framework is presented in Fig. 11. It is designed to be used as a guide for future replication studies, particularly for the health research in mind.

Step 1 and 2 in this framework address the need for awareness of the study characteristics in both the original and comparator studies. These differences can explain reduced replicability performance. We also highlight the need to perform internal validity and stability of clustering model performance at the individual study level. All clustering algorithms will generate clusters but the quality of these clusters must be evaluated. We suggest internal stability assessment across slightly perturbed datasetsc (by bootstrapping) and/or using different model initialisations on the same dataset. This allows assessment of the cluster stability across variations in both the model and the dataset.

In the absence of available external datasets, as in the case of the work here, these measures can provide a means of assessing model stability. Ideally however comparator study datasets would be available to the researcher for cross-study cluster analysis replicability. This is not always the case with sensitive data such as health data. A recent publication provides a novel method for this procedure^30^.

## Discussion

Based on analysis of the largest audiogram dataset in the UK, there is support that cluster analysis using the GMM can identify audiogram-defined subtypes in patients with SNHL that are partially similar to those found in a different patient sample from the USA^19^. Discrepancies between the 2 models can be accounted for by differences between the patient populations (age, sex and disease severity), difference between study protocols and limitations in the stability of the GMM across datasets. These differences and their impact on the model outcomes illustrate potential pitfalls in machine learning research practice that need to be overcome to ensure the safe and generalisable translation of these promising methods into clinical practice. We present a clustering replication framework to aid the researcher in addressing these areas. The framework highlights the importance of thoroughly describing the study population thus enabling other researchers to assess the comparability of their own datasets and consider how differences may influence clustering results. The framework also underscores the critical importance of validation, with internal validation as a necessary step to assess the stability and robustness of clustering within the study sample, and external validation—where feasible—used to evaluate the generalizability of findings to other populations.

Our study identified 9 distinct audiogram phenotypes in comparison to the 10 in the MEE dataset. These 9 profiles can be assigned into five broader “super clusters”, where each super cluster represents a shared audiometric profile with varying severity levels. Subtype 9 represents individuals with normal hearing. A presbyacusis-like profile, characterized by normal low-frequency hearing with varying degrees of high-frequency hearing loss, was observed in Subtypes 4 and 7. Flat low frequency and down-sloping high frequency hearing loss profiles were identified in Subtypes 5 and 6. A down-sloping hearing loss pattern affecting all frequencies was evident in Subtypes 1, 2, and 8, indicating a progressive decline across the audiometric spectrum. Lastly, Subtype 3 exhibited a notched profile with severe thresholds. Despite identifying nine distinct clusters, the observed similarities in audiometric features that allow grouping into five super-clusters suggests that some clusters may reflect different stages within a shared disease process rather than distinct diagnostic categories.

Broadly, the identified audiometric profiles across both studies display a downward sloping profile which manifests as high-pitched hearing loss^31^. This is the most common hearing loss pattern and pathologically is secondary to cochlear dysfunction at the level of the sensory hair cells due mostly to aging, but also noise trauma and genetic predisposition^32^. Both the RNENT and MEE datasets were over-represented by older patients, which mirrors the user profile for hearing loss services in both the UK and the USA^33^.

In this study, we were not however able to replicate all the audiogram profiles found in the MEE study. We were unable to identify the notched audiogram profile classically associated with NIHL. Conversely our implementation of the GMM identified novel profiles not found in the MEE study.

Differences in age, disease severity and sex distribution between the two study populations likely contribute in part to the observed variations in cluster solutions. The RNENT study included a higher proportion of older patients, with 71% over 50 years, compared to 63% in the MEE dataset. Additionally the MEE dataset had an upper age limit of 80 compared to 100 in the RNENT dataset. The decision not to restrict the upper age limit in the RNENT study to mirror the MEE study was made to accurately reflect the clinical population treated at our unit. Older patients tend to have worse hearing thresholds across frequencies, and with longer-standing hearing loss conditions, early audiometric characteristics (such as the noise-induced hearing loss characteristic 4 kHz) may be lost over time^34,35^. 

Additionally, the RNENT population had less restrictive audiometric inclusion criteria compared to the MEE dataset, resulting in a cohort with more severe hearing loss, particularly at low frequencies. The 2 clusters (3, 6) that could not be matched to the MEE model both are characterised by having the highest thresholds at low frequencies across all 9 audiogram profiles.

Sex distribution also differed between the populations, with women overrepresented in the RNENT study. Sex-related differences in hearing loss pathologies and the loss of oestrogen-protective effects in older women may further contribute to variations in disease severity profiles between the two datasets^36,37^. Our eldest age groups are over-represented by women who may have lost the oestrogen-protective effects at this time.

A further contributing factor is the choice of which audiogram to include for patients with multiple tests. Unlike the MEE study, which selected the first audiogram, we chose a random audiogram per patient in those patients with repeat measures. This choice facilitates representation of hearing loss across the disease trajectory, avoiding bias towards early stages which may also explain the differences in severity profiles of the identified clusters between the two studies.

These differences and methodological decisions underscore the need for rigorous validation to assess the stability, robustness, and generalizability of clustering models across diverse populations and settings. In the absence of definitive ground truth, model outcome evaluation is a challenges in both our study and its’ US counterpart. Assessing the model’s ability to identify reproducible clusters in independent datasets strengthens the evidence that the observed clusters are not due to dataset-specific artifacts but reflect real, underlying biological phenomena. However, the sensitive nature of health data often restricts data access, limiting such external validation and replicability assessments between studies to qualitative assessments only as was the case here.

Using publicly available datasets like NHANES to identify SNHL subtypes offers a solution to data access issues. However, NHANES primarily includes AC measures, with BC measures recorded only in the 1974 study year. This poses a significant issue as relying solely on AC thresholds for patient selection prevents distinguishing between SNHL, conductive hearing loss, or mixed patterns. These conditions reflect fundamentally different sites of lesion, and clustering without making this distinction risks grouping together clinically and pathologically distinct entities. Whilst the NHANES dataset does include additional measurements of otoscopy and tympanometry that can be used to aid in differentiating between SNHL and CHL, this is not definitive. Given the high stakes of AI applications in healthcare, where errors can directly impact patient safety and treatment decisions, it is essential that models are developed using high-quality, well-characterized data. It would however be valuable to investigate how well otoscopy and/or tympanometry can distinguish CHL from SNHL when only AC thresholds are available, potentially facilitating the dataset’s use in future AI studies. Currently, no other publicly available audiogram datasets exist to our knowledge. For instance, UK Biobank only contains a large repository of speech-in-noise tests.

Alternative emerging approaches to circumvent data access issues include federated learning and generation of simulated/synthetic datasets^38^. Synthetic datasets show a small decrease in model accuracy in supervised machine learning problems than models trained on real data however studies evaluating their generalisability within UML paradigms are limited^39^. However, there is promising evidence that synthetic datasets can be created that balance risk of private information leakage and maintaining the statistical properties of the original datasets^40^.

To address data access limitations here, we employed alternative evaluation methods, emphasizing the internal stability of our clustering results to assess their generalizability and validity as a proxy. Although the GMM displayed consistent clusters across different initializations, its performance diminished with minor dataset perturbations, raising concerns about the robustness and reproducibility of the identified subtypes. The variability in cluster allocations observed through bootstrapping suggests that the model’s outcomes may be unstable and overly sensitive to specific data points, rather than capturing truly inherent, reproducible hearing loss phenotypes. This variability is particularly concerning in a clinical context, where unstable classifications could lead to inconsistent patient stratification and affect the generalizability of findings to new populations.

Beyond issues of statistical robustness and methodological considerations, the clinical significance of the identified subtypes must be carefully evaluated. A key question is whether the nine clusters identified in our analysis correspond to recognized clinical subtypes that align with established medical knowledge and clinical experience. While cluster analysis is a powerful tool for knowledge discovery, its clinical utility depends on whether the identified clusters represent meaningful phenotypes rather than dataset-specific artefacts.

In our study, several subtypes appeared to have clear clinical relevance, including normal hearing, profiles consistent with presbycusis, and more severe stages of SNHL. To robustly establish these clusters as clinically meaningful phenotypes, additional validation is necessary. This could be achieved by demonstrating that each subtype is associated with a measurable clinical endpoint, such as disease prognosis, treatment response, or correlation with independent biomarkers (e.g., imaging data, genetic markers). Such validation would strengthen the argument that these clusters are not just statistical constructs but instead represent biologically and clinically distinct hearing loss subtypes with real-world implications for diagnosis and management.

Furthermore, our analysis did not identify certain audiometric profiles, such as the reverse-sloping and 4 kHz notch patterns, which are recognized in established clinical classifications. We argue that this reflects limitations in the clustering model, particularly in capturing audiometric subtypes that are clinically meaningful but may be less statistically dominant. To explore this further, future work could consider alternative models or validation approaches that successfully distinguish these subtypes, thereby confirming whether the absence of these profiles is truly a methodological limitation or a reflection of the dataset’s underlying structure.

This study in not without limitations. Firstly, we acknowledge that use of audiograms to characterise the hearing loss profiles represents a pragmatic choice. While audiometry remains the most widely performed investigation for hearing loss, it does not capture all perceptual aspects, particularly suprathreshold deficits. Whilst we advocate for a more comprehensive assessment involving multiple tests^41,42^, the study aligns with current clinical practice to ensure relevance and applicability to a broader patient population. Incorporating additional hearing assessments into UMLs is limited by the small number of patient numbers receiving these specialized tests, reducing the clinical utility of models intended for real-world application^43^.

Additionally, while analysing each ear separately aligns with the methodology of the MEE study and allows for direct cross-study comparison, it introduces certain limitations. Given that individuals have two ears, hearing loss is often correlated between them due to shared genetic, environmental, and physiological factors. Treating each ear as an independent observation ignores this inherent dependency, potentially leading to an overestimation of sample size and an underestimation of within-subject variability. To account for this, a supplementary analysis was conducted to examine the correlation between subtypes across paired ears. This revealed high correlation in only three phenotypes, suggesting that while some subtypes exhibit strong bilateral similarity, the majority may follow more distinct patterns, hereby mitigating concerns about treating each ear separately.

Future research should also explore additional population characteristics beyond age and sex, including under-studied risk factors such as social, nutritional, and health factors during childhood, genetic susceptibility, noise and ototoxic drug exposure, and infection. Enriching patient feature space with relevant biomarkers could enhance understanding and characterization of hearing loss patterns.

In conclusion, we have used the GMM to identify 9 audiometry-defined clusters that partially overlap with those identified within an external dataset. Differences in study design including inclusion criteria contribute to the incomplete replicability between the 2 studies. The performance of this algorithm is observed to be constrained in generating consistent clusters across multiple runs, especially in the presence of noise in the dataset. This raises concerns about its performance across diverse datasets. For clustering to inform clinical workflows, research practices need to be elevated against higher levels of success than identification of clusters. Adhering to the principle of “First do no Harm,” methods must demonstrate safety in patient use^44^. Models should exhibit generalizability and replicability across diverse datasets, as limited applicability to the training population undermines clinical utility. In the context of cluster analysis for disease phenotyping, a rigorous quality analysis is essential, involving performance against benchmark data or, when unavailable, quantitative measures of replicability. We have created a framework to facilitate better research practice. Lastly, we emphasize the imperative for establishing a purpose-built audiometric public dataset and fostering greater sharing of local datasets.

## Methods

### Setting and study population

Data from patients aged 18–100 years who underwent pure tone audiometry (PTA) at the Royal National Ear, Nose and Throat (RNENT) Audiology Department between 1981 and 2021 were used. The RNENT is the only hospital dedicated to the management of ENT problems in Europe and sits within University College London Hospital (UCLH) National Health Service (NHS) Trust. During the study period, we acknowledge that audiometric equipment will have changed however this is mitigated by the introduction of audiometric equipment calibration which became standard practice at the start of this period^45^. Results were compared with published data from the Massachusetts Eye and Ear (MEE) study^19^. The study inclusion criteria, alongside that of the MEE study, are summarised in Table 3.

**Table 3:** Comparison of study inclusion criteria, exclusion criteria and protocol deviations between the Royal National ear, nose and throat study (RNENT) and the Massachusetts eye and ear (MEE) study.

RNENT study	MEE study
**Inclusion criteria**	
Age: ≥18 Test frequencies: 0.25, 0.5, 1, 2, 4 and 8 kHz. Other: Completed values for sex and date-of-birth	Age: 18–80 Test frequencies: 0.25, 0.5, 1, 2, 3, 4 and 8 kHz. (3 kHz was imputed)
**Exclusion criteria**	
CHL: ≥25dB air-bone gap at 2 frequencies between 0.5, 1, 2 kHz	CHL: ≥ 20dB air-bone gap at 1 frequency or ≥ 15dB at 2 consecutive frequencies Severity: ≥ 85dB thresholds at or below 2 kHz
Protocol	
Hearing test selection: Random Ear: Left and right per patient	Hearing test selection: The first hearing test Ear: Left and right per patient

CHL, conductive hearing loss; dB, decibels.

### Model input

The feature space for the model was air-conduction (AC) thresholds measured in decibels (dB) across 6 test frequencies (0.25, 0.5, 1, 2, 4 and 8 kHz). These thresholds were chosen in line with the British Society of Audiology’s recommended procedure for pure-tone AC and bone-conduction (BC) threshold audiometry (Appendix 1)^46^. Unlike, the MEE study we did not include 3 kHz in our analysis as this is not measured in standard protocol in the UK and would therefore need to be imputed. Imputation can lead to over-estimated precision in the imputed values as these values have no error term in their estimation^44^.

Audiograms from patients with SNHL of any cause were included in the study. SNHL was identified based solely on audiometric criteria by excluding any records that showed evidence of conductive hearing loss (CHL). CHL was defined as an air-bone gap (difference between AC and BC thresholds) of 25 dB at two or more of the following frequencies: 0.5, 1, and 2 kHz in line with guidance from the British Society of Hearing Aid Audiologists (Appendix 2)^44,47^. This standard differs from that used in the MEE study (Table 3) but aligns again with local procedure.

A single audiogram was used per patient. This was chosen randomly to not bias the dataset towards patients with earlier stages of hearing loss. In contrast the MEE study selected the first audiogram. To maintain methodological consistency with the MEE study to facilitate robust cross-study comparison, the audiogram of each ear per patient was treated as an independent observation.

### Databases

The dataset was created through data linkage across 3 different databases: Auditbase, EPIC and Archived Data Storage (ADS).

#### Auditbase

This is a clinical management system used by the RNENT Audiology Department to conduct hearing testing. The audiogram results, patient sex and patient age were extracted from Auditbase.

#### EPIC

EPIC is the electronic health record system used at UCLH since 2019. Sex and age data were not always reliably completed within the Auditbase database. Data linkage was performed between Auditbase and EPIC to gather missing sex and age information.

#### ADS

Historic patient records that pre-dated the arrival of EPIC within the hospital are stored in ADS therefore patients with missing demographic details that were not found in the EPIC database were linked to ADS.

### Ethics

The study was approved by the UCLH Data Trust Committee (DAC) under the Data Access Process for Research (DAP-R). This process devolves ethical approval for data-only studies that require access to routinely collected anonymous data and has been approved by the South West - Central Bristol Research Ethics Committee under IRAS ID 299,136. De-identified data was used in this study, data was analysed retrospectively and all data were collected as part of the delivery of routine hearing health care. As such, in line with the UK Common Law of Confidentiality and Consent, informed consent was not required. The research was performed in accordance with relevant guidelines and regulation.

### Data pre-processing

Audiogram data from Auditbase is structured in a tabular format, where each row represents the hearing thresholds from a single patient during one hearing test, specific to the method used to obtain those thresholds. The two main methods are AC, which involves playing tones directly into the ear, and BC, where tones are delivered to the temporal bone. As a result, a single hearing test may be represented by multiple rows—each row corresponding to a distinct set of thresholds measurement for a given method. For clarity, we refer to each of these rows as a *curve* throughout this work. Routine testing may involve AC only, or both AC and BC measurements. To identify complete hearing tests in the database, AC and BC thresholds from the same test were merged, resulting in each hearing test being represented as a single row. Data cleaning was performed both before and after joining AC and BC records to ensure the integrity of the final dataset. Pre-join cleaning prepared individual records for accurate merging and ensured that only complete entries were included. Post-join cleaning then filtered the dataset to retain a single hearing test per patient per day, with valid threshold values indicative of SNHL (see Appendix 4 in Supplementary Materials for a complete description).

In brief, prior to the join, duplicate curve records were removed. For Auditbase curve records with missing values for sex and age, cross-linkage between Auditbase and ADS and Auditbase and EPIC was performed to retrieve these values where they existed. If these were missing then the curves were removed. Audiogram curve records with no threshold values (i.e., “empty” records) were removed. Duplicate audiogram curve records—defined as records indexed as separate audiograms but conducted on the same day with the exact same thresholds —were also removed. In cases where multiple, non-duplicate audiograms existed for the same day, a single audiogram curve record was included only based on a predefined rule (outlined in Appendix 4 in Supplementary Materials).

After audiogram curve records were joined, audiogram records with threshold values outside the standard testing range (−10 to 120 dB) or not recorded in multiples of 5 dB were removed, as these do not conform to routine audiometric testing protocols. Only records meeting the criteria for bilateral SNHL, as defined in Table 3, were included (see Appendix 4 for complete breakdown). This group was further filtered to only include only patients aged ≥ 18. Finally a single random audiogram was included per patient for patients with multiple audiograms in the database.

### Gaussian mixture model

The GMM is a generative probabilistic approach that models a dataset as a combination of multivariate Gaussian distributions, each with its own unknown mean and covariance^48^. While often used for density estimation, GMMs are also commonly applied as UML methods to cluster data points that likely originate from the same underlying distribution. In this context, each audiometric phenotype corresponds to a multivariate Gaussian distribution over six-dimensional vectors, where each vector represents an individual audiogram defined by threshold values at six standard test frequencies.

GMM uses the expectation-maximisation (EM) method for estimating its parameters (the means, covariances and weights). These model parameters can be initialised in several ways. We used k-means to initialise these parameters to mirror the approach of the MEE study to act as a direct comparison. These initial parameter estimates are iteratively improved by alternating between an expectation (E)- step where expectations of the log-likelihood function for each data point are computed using the current parameters, and then the maximisation (M)-step where the means for each gaussian are updated based on the maximum likelihood estimate. This process is repeated until convergence is achieved. The convergence criterion was set to 1e-3. We were unable to achieve convergence with the lower limit used in the MEE study of 1e-6.

The GMM requires setting the number of clusters in advance. A systemic iterative approach was used to select the optimum cluster number using the Bayesian Information Criterion (BIC) and the Akaike Information Criterion (AIC). The BIC assesses the negative log-likelihood of the model with a small BIC indicating good model fit. The BIC has the advantage over the AIC because the latter tends to overfit the data and select more complex models^49^. The model was run iterating through cluster numbers 2 to 15 (these numbers were selected to mirror the MEE study). As the GMM is non-deterministic and sensitive to the starting points, the model was run from 21 different random seeds leading to 294 combinations of cluster number and random seed models. The mean BIC and AIC were calculated for each cluster number across all 21 seeds and these values plotted against cluster number. The elbow method was used to visually determine the optimal number of clusters^50^.

The GMM takes several other parameters. Covariance type controls the degrees of freedom in the shape of each cluster. The regularization parameter is added to the diagonals of the covariance matrices to ensure they are positive, thus avoiding ill-conditioned covariance matrices. For consistency we chose the same values for these 2 parameters as the MEE study: Full covariance type and the regularisation parameter 0.01. We did, however, also perform additional analysis through a GridSearch approach iterating through the 4 different covariance types (Full, Diagonal, Tied, Spherical) and a range of regularisation parameters ((0.001–0.01 in increments of 0.001) with best performance converging on our final parameter values (see appendix 3).

Each identified cluster was characterized by its audiometric profile—summarized using the mean and standard deviation of hearing thresholds at each test frequency—and by the demographic features of its assigned patients, including mean age (± standard deviation) and sex distribution (proportion of males and females).

As each ear was treated as an independent observation, an additional analysis was conducted to assess the correlation between subtype allocation between paired ears. This was performed to assess for a potential dependence between ears. Between ear symmetry was assessed by calculating the conditional probabilities of cluster membership of the right ear given the cluster membership of the left ear. This was performed for direct comparison of the MEE study results which performed this analysis, as well as exploring whether treating 2 ears from the same patient as independent observations is valid.

### Identification of known clinical audiogram profiles

To determine whether the identified clusters capture clinically-established audiogram patterns, we examined the presence of two exemplar SNHL profiles in our dataset: noise-induced hearing loss (NIHL), characterized by an inflection at 4 kHz^28^, and low-frequency hearing loss (also called reverse-slope hearing loss), typical of Ménière’s disease^29^.

NIHL is the 2nd most common cause of hearing loss and results from exposure to noise sufficient in intensity to cause hearing loss, typically sustained in an occupational setting. NIHL has a characteristic audiogram pattern associated with a drop in hearing at 4 kHz followed by a recovery, commonly referred to as a notch. This notch is more prominent at the early stages of hearing loss and becomes lost over time as hearing thresholds worsen. We identified whether there were any audiograms displaying the 4 kHz audiometric notch in our dataset. Coles’ criterion was used to define and identify audiograms with the audiometric notch characteristic of NIHL. This is defined as at least a 10dB increase in hearing threshold at 4 kHz compared to 1–2 kHz and 8 kHz^28^.

Reverse-slope hearing loss was defined as an average reduction of 10dB in the high frequencies (4 kHz and 8 kHz) compared to the low frequencies (0.25 kHz and 0.5 kHz)^29^.

### Replication studies

In the absence of access to the MEE dataset, we could not directly assess whether the clusters we identified were replicable in their dataset. The analysis was limited to comparing both the number of clusters and the audiogram profiles of identified subtypes between the two studies. To evaluate the stability of our subtypes quantitatively we examined whether similar cluster structures emerged under perturbations to the data (see the *Across different bootstrap samples* subsection below) and model (see the *Across different initialisations* subsection below)^26^.

The Jaccard coefficient, a similarity measure between sets, was used to evaluate cluster stability across different initializations and bootstrap samples. It is calculated by taking the intersection of elements within two sets and dividing it by the union of all unique elements in both sets. A Jaccard score of 1 indicates a perfect match whereas a score of 0 indicates no similarity or overlap between the sets. Further details can be found in the original citation^51^. Originally implemented in R as the clusterboot package, we developed a Python version, accessible through the code repository.

#### Across different bootstrap samples

Bootstrapping generates resampled datasets (with replacement) of the same size as the original, introducing stochastic variation of the dataset while preserving overall structure.

For detailed description of the bootstrap method please see Hennig (2007)^51^. 1000 bootstrap samples, each of equal size to the original dataset, were drawn with replacement from the original dataset. For each bootstrap sample, clustering was performed using a GMM, with the random seed chosen based on the lowest AIC value in the original model. The Jaccard coefficient was then computed in a cluster-wise manner for data points present in both the original dataset and the bootstrap sample, by identifying the most similar cluster in the bootstrap sample for each original cluster and assigning calculating the Jaccard coefficient. The mean Jaccard score was calculated across all bootstrap samples^51^.

#### Across different initialisations

EM, which underpins the GMM, is sensitive to initial random seed values. Different initializations can lead EM to converge to different local optima, potentially producing distinct clustering outcomes.

The GMM model was initialized 20 times using different random seed values, while all other hyperparameters were held constant to match those of the main model. The random seed chosen for the main model was excluded which is why there were 20 different random seeds rather than 21. The Jaccard coefficient was then computed comparing the cluster assignment of audiograms in the original model to those generated for the different initialisations in a cluster-wise manner identifying the most similar cluster to the original clusters and assigning it a Jaccard coefficient.

#### Impact of data quantity on replicability

To evaluate how well the clustering structure is preserved at different sample sizes, we systematically sampled the original dataset to create nine subsets, ranging from 10% to 90% of the full dataset. For each subset, we ran the original GMM and compared the cluster assignments with the resulting cluster labels from running the GMM on the entire dataset using the Jaccard score.

### Clustering replication framework

Based on insights gained through this project, we developed a framework to support researchers aiming to replicate clustering-based analyses from previous studies. Our goal was to address the current lack of structured guidance for performing clustering with replication in mind—something we found would have been valuable during our own work. While the framework was developed with health data science research in mind, its principles are broadly applicable across disciplines where clustering is used.

### Statistical analysis

Descriptive statistics were produced to describe the study population and identified clusters in terms of age, sex and presence of existing audiogram types. Qualitative methods were used to compare the cluster outputs between the 2 datasets given the MEE dataset is not publicly available and access was not provided on request.

## Supplementary Information

Below is the link to the electronic supplementary material.

Supplementary Material 1

Supplementary Material 2

## Data Availability

The threshold data and patient demographics are sourced from patient electronic health records at University College London Hospital (UCLH) (see *Databases* subsection of Methods). The data are accessed via formal request to the Information Governance team at UCLH. This access can only be requested by UCLH staff and must be made through a request portal (Data Explorer - DEX) that can only be accessed within the UCLH NHS firewall. This process complies with local ethical and legal requirements underpinned by the local Information Governance framework. Further information about this process can be found: https://www.uclhospitals.brc.nihr.ac.uk/data-explorer-dex.

## Code availability

The PostGres SQL scripts for the data pre-processing and the Jupyter Notebook containing the python code for model development and implementation are available from https://github.com/lildim22/Audiogram_GMM_RNENT. All code supporting the findings of this study has also been deposited in Zenodo and is accessible via the DOI: 10.5281/zenodo.17673967.
