# Supplementary material for: Identification of sensorineural hearing loss subtypes using unsupervised machine learning and assessment of their replicability
Source: Sci Rep. 2026 Jan 20;16:3774. doi: 10.1038/s41598-025-33815-9 (PMC12852672; doi:10.1038/s41598-025-33815-9)
Supplement: Supplementary file 1 — Supplementary Material 1 [file 41598_2025_33815_MOESM1_ESM.docx]

# Supplementary Materials

## Appendix 1

Table displaying criteria for air-conduction and bone-conduction testing taken from the British Society of Audiology’s recommended procedure for pure-tone AC and bone-conduction (BC) threshold audiometry

|  | **Air-conduction** | **Bone-conduction** |
| --- | --- | --- |
| Test frequencies | 0.25 kHz, 0.5 kHz, 1 kHz, 2 kHz, 4 kHz, 8 kHz | 0.25 kHz, 0.5 kHz, 1 kHz |
| Additional notes^1^ | All of the above frequencies should be tested as minimum.  Where needed and practicable, test also at intermediate frequencies 750 Hz, 1500 Hz, 3000 Hz and 6000 Hz. | It may not be necessary or appropriate to test at all these frequencies in every case |

## Appendix 2

Table displaying criteria used for identifying excluding CHL audiograms taken from British Society of Hearing Aid Audiologists^2,3^

|  | **Conductive Hearing loss** |
| --- | --- |
| Official criterion: | 25 dB or greater air-bone gap present at two or more of the following frequencies: 500, 1000, 2000Hx or 4000 Hz |
| Adapted criterion: | However: updated guidance for bone-conduction testing procedure in Appendix 1 states not all these frequencies need to be tested.  Therefore the above criterion was adapted to included 2 or more frequencies with an air-bone gap of >25dB between 0.25 kHz, 0.5 kHz, 1 kHz |

## Appendix 3

Table displaying parameters used for tuning the GMM model

| **Parameter** | **Value** |
| --- | --- |
| Random state | 1, 10, 20, 30, 40, 50, 60000, 70, 80, 9333330, 100, 110, 120, 13044, 140, 150, 160, 170, 18024, 190, 200 |
| Regularisation value | 0.001, 0.002, 0.003, 0.004, 0.005, 0.006, 0.007,  0.008, 0.009, 0.01 |
| Cluster number | 2, 3, 4, 5, 6, 7, 8, 9, 10, 11, 12, 13, 14, 15 |
| Covariance type | Spherical, tied, diagonal, Full |
| Convergence threshold | 1e-3 |

## Appendix 4

Identifying patients with sensorineural hearing loss (SNHL) using audiogram data requires multiple stages of data pre-processing. This section is intended to be read alongside Figure 1 and the Supplementary Figure.

In Auditbase, audiogram data is stored in a tabular format, with each row representing a set of thresholds collected from a patient using a specific test modality. Audiograms are most commonly conducted by measuring air-conduction (AC) thresholds—where tones are delivered via headphones—or by measuring both AC and bone-conduction (BC) thresholds, with BC tones transmitted through the temporal bone. When both AC and BC thresholds are measured during a single test, Auditbase stores them as two separate records (termed "curves") in the AUDIOGRAMCURVE table. These paired curves must be joined to reconstruct a complete audiogram that contains both AC and BC data.

The initial filtering step limited the dataset to AC and BC curve records, yielding 349,060 individual curves. Further filtering was carried out at two stages: **pre-join**, where curves were assessed for completeness and suitability (e.g., excluding invalid or partial tests), and **post-join**, after curves belonging to the same audiogram had been merged into a single record. This two-step filtering process ensured that the final dataset comprised valid, interpretable audiograms suitable for identifying SNHL.

Pre-join

- The initial filtering step involved the removal of duplicate audiogram curve records across all column values (yielding N = 348851).
- Records with incomplete or nonsensical values for sex (values that were not male or female) and date-of birth (values that predated 01/01/1901 or where date-of-birth was after the date of the audiogram) were subsequently excluded. Prior to removal, these fields were checked for both completeness and validity. Where values were missing, patient records were cross-referenced with electronic health record systems—EPIC for records post-2019 and ADS for those prior to 2019—and updated where matches were found. This yielded N = 332229 records.
- The next step records were removed which had no audiogram thresholds across the 6 test frequencies in both ears recorded (this step is described as the removal of empty curves). Essentially these rows had patient details and audiogram date but no measured thresholds. This yielded N = 314712 curves.
- In the next step, we removed patients who had multiple audiogram curves of the same type (AC or BC) recorded on the same day with identical threshold values across all frequencies. These curves differed only by the audindex field, which tracks the sequence of audiograms for a given patient on a given date. In other words, some individuals had repeated identical curves within a single day. Duplicate curves were removed, resulting in a dataset of *N* = 301,546.
- To address cases where patients had multiple non-duplicate audiograms recorded on the same day, only a single audiogram was retained—this step corresponds to the “removal of multiple entries” in the Supplementary Figure. This ensured that only one audiogram per patient per date was included, consistent with typical clinical practice in which hearing tests are not routinely repeated on the same day unless prompted by a specific issue (e.g., need to re-test certain frequencies or re-assess after an intervention such as cerumen removal). A heuristic was applied to determine which audiogram to retain. Among multiple audiograms recorded on the same day, the audiogram curve with the fewest missing values across the six key test frequencies was selected. In cases of ties—i.e., when multiple curves had the same number of complete thresholds—the most recent curve (determined by the highest audindex value) was retained, assuming it reflected the final and most accurate measurement of the day. This process yielded *N* = 295,913 audiogram curve records.

**Join**

To obtain a complete audiogram for a patient, all AC and BC curves from a single test session were joined using the primary key that identifies each audiogram. This join operation yielded three distinct groups:

1. Patients with BC-only data (*N* = 344).
2. Patients with AC-only data (*N* = 52,614)
3. Patients with both AC and BC thresholds recorded in a single audiogram. 242,956 individual curves were successfully paired to form *N* = 121,478 complete audiograms

These 3 different categories are considered in turn in the post-join subsection below.

**Post-join**

Data pre-processing primarily involved two stages: filtering to exclude records with values that do not conform to routine audiometric testing protocols (values must be multiples of 5 and within -10 to +120 dB), and generating a subset of patients with SNHL.

All audiograms with evidence of SNHL in at least one ear were initially identified (see SQL code in the GitHub repository). However, as this study focused on phenotyping patients without an ABG, only those with bilateral SNHL—defined as the absence of an ABG in both ears—were included in the final dataset. While the SQL script generates a broader SNHL database that includes unilateral cases, only audiograms with bilateral absence of ABG were retained for analysis and this process is described here.

1. Those with BC only (n = 344)

BC without AC is of limited utility clinically, and as such this group were not used.

1. Those with only AC (n = 52614)

The first step in this branch involves only including audiogram thresholds with possible values. Audiogram thresholds must fall within the range of -10 to + 120dB and can only take values that are multiples of 5. This step was performed after the join to avoid the situation where audiogram curves forming part of an audiogram were removed. This could, for example, lead to a situation where someone appears to have only AC measured when in reality they had AC and BC measured but the BC curve was removed before the 2 curves were joined. This yielded N = 52433 audiogram records.

In the presence of hearing loss, AC without BC cannot tell us whether a person has a SNHL or CHL in general. However there are rules-of-thumb where this may occur. Firstly if someone has normal hearing thresholds, BC is not typically performed. Secondly, if someone has unchanged AC thresholds and they previously had no ABG then bone conduction is not repeated. All patients in the AC only group were also present in the AC/BC group therefore they were considered to fall into 2^nd^ category and were kept in the dataset.

This subgroup was then broken down into groups with threshold measurements for the 6 test frequencies in both ears (N = 41522), left ear (N = 1746), right ear (N = 1600) and neither ear (referred to as incomplete in Supplementary Figure, N = 7565). In this study we were interested in patients with bilateral SNHL so only those with SNHL in both ears (N = 41522) were kept.

1. Those with Both AC and BC Thresholds (N = 121,478)

As described earlier, this group was formed by joining AC and BC curves conducted on the same day for the same patient to reconstruct complete audiogram records. As with the AC-only group, we retained only audiograms with plausible thresholds—defined as values within the measurement range of –10 dB to +120 dB and in 5 dB increments—yielding N = 121,218 audiogram records.

This dataset was further filtered to include only audiograms with complete AC threshold data for the six standard frequencies (0.25–8 kHz) in both ears (N = 114,146).

Among audiograms with complete AC thresholds in both ears (N = 114,146), the next step in the data flow, shown in the Supplementary Figure, splits based on the extent of BC testing:

1. BC performed in both ears: N = 27,512
2. BC performed in the left ear only: N = 39,711
3. BC performed in the right ear only: N = 43,106
4. BC Performed in Both Ears

Patients in this group were assessed for SNHL using the criteria outlined in Appendix 2. In brief, SNHL was defined by the absence of ABG ≥25 dB at two or more frequencies among 0.25, 0.5, and 1 kHz.

The ABG was calculated by subtracting the BC threshold from the corresponding AC threshold. When both masked and unmasked BC thresholds for a given ear were available, masked thresholds were preferred due to their greater accuracy in isolating cochlear function.

A small subset of patients were classified as “incomplete” (Supplementary figure) if they only had BC for two of the three frequencies and one of those yielded an ABG and the other did not. Since they did not meet the CHL criterion (i.e., an ABG >=25 dB at two or more frequencies) they were still eligible for inclusion in the SNHL cohort. This group (complete + permissible incomplete) yielded **N = 17,586** SNHL audiograms.

1. BC Performed in One Ear Only

For audiograms with BC thresholds measured in only one ear (left or right), an additional distinction was made based on the use of masking:

- Masked BC thresholds can only be used to determine the ABG in the ipsilateral ear.
- Unmasked BC thresholds may be used to estimate the ABG in both ears.

*Unilateral unmasked +/- masked BC*

When both masked and unmasked thresholds were available for the same frequency, masked BC thresholds were used to determine the ABG for the ipsilateral ear, while unmasked BC-thresholds were used to determine the ABG for the contralateral ear. Otherwise if only unmasked BC was performed this was used to determine the ABG in both ears.

Following the same SNHL criteria as above, patients with:

- No ABG in both ears,
- No ABG in one ear and incomplete data in the other, or
- Incomplete in both ears but no CHL pattern

were included. This yielded:

- N = 24,777 for audiograms with left-ear BC
- N = 25,493 for audiograms with right-ear BC

*Unilateral masked BC*

Importantly, where only masked BC thresholds were available for a single ear, these cases could not be used to assess SNHL in the contralateral ear and were therefore excluded from the dataset as the dataset was filtered to include only bilateral SNHL audiograms.

From the original 121, 478 audiograms with both AC and BC thresholds, 67,856 were retained.

**Final dataset**

After the process of including only audiograms with SNHL, there were 109, 378 audiograms in the dataset. These were filtered to only include patients age 18 or older (N = 95253) and finally a random audiogram per patient was selected yielding the final number of audiograms 54927. As each ear was considered separately, the left and right ear threshold values were separated yielding a total of 109854 audiograms from a single ear.

## References

1 British Society of Audiology. *Recommended procedure: Pure-tone air-conduction and bone conduction threshold audiometry with and without masking*, 2018).

2 Audiology, B. A. o. *Onward Referral Guidance for Adult Audiology Patients*, <<https://www.baaudiology.org/app/uploads/2019/07/BAA_Guidance_for_Onward_Referral_of_Adults_with_Hearing_Difficulty_Directly_Referred_to_Audiology_2016_-_minor_amendments.pdf>> (2017).

3 Audiology, B. S. o. *Recommended procedure: Pure-tone air-conduction and bone conduction threshold audiometry with and without masking*, 2021).
